# The Pollen Donor Affects Seed Development, Taste, and Flavor Quality in ‘Hayward’ Kiwifruit

**DOI:** 10.3390/ijms24108876

**Published:** 2023-05-17

**Authors:** Yanan Chai, Weijin Hong, Hang Liu, Xia Shi, Yanfei Liu, Zhande Liu

**Affiliations:** College of Horticulture, Northwest A&F University, Yangling 712100, China; yananchai@nwafu.edu.cn (Y.C.); hongweijin1997@163.com (W.H.); a417983954@126.com (H.L.); sxpddsx@163.com (X.S.); lyfkiwi@nwafu.edu.cn (Y.L.)

**Keywords:** kiwifruit, pollen donor, seedless, fruit quality

## Abstract

To investigate how different species or ploidy level of pollen donors affects the fruit quality of kiwifruit, flowers of ‘Hayward’ kiwifruit (a hexaploid *Actinidia deliciosa* cultivar, 6x) were hand-pollinated with pollen from ten different male donors. Kiwifruit plants pollinated with four distant species—M7 (2x, *A. kolomikta*), M8 (4x, *A. arguta*), M9 (4x, *A. melanandra*), and M10 (2x, *A. eriantha*)—had a low fruit-setting rate and therefore were not investigated further. Of the other six treatments, kiwifruit plants pollinated with M4 (4x, *A. chinensis*), M5 (6x, *A. deliciosa*) M6 (6x, *A. deliciosa*) had a larger fruit size and weight than those pollinated with M1 (2x, *A. chinensis*) and M2 (2x, *A. chinensis*). However, pollination with M1 (2x) and M2 (2x) resulted in seedless fruits, having few small and aborted seeds. Notably, these seedless fruits had higher fructose, glucose, and total sugar and lower citric acid content. This resulted in a higher sugar to acid ratio compared to fruits from plants pollinated with M3 (4x, *A. chinensis*), M4 (4x), M5 (6x), and M6 (6x). Most volatile compounds increased in the M1 (2x)- and M2 (2x)-pollinated fruit. A combination of principal component analysis (PCA), electronic tongue, and electronic nose suggested that the different pollen donors significantly affected the kiwifruit’s overall taste and volatiles. Specifically, two diploid donors had the most positive contribution. This was in agreement with the findings from the sensory evaluation. In conclusion, the present study showed that the pollen donor affected the seed development, taste, and flavor quality of ‘Hayward’ kiwifruit. This provides useful information for improving the fruit quality and breeding of seedless kiwifruit.

## 1. Introduction

Kiwifruit is a dioecious plant, and it must be cross-pollinated. The phenomenon of the pollen genotype’s direct effect on the seed and fruit development and characteristics between fertilization to seed germination is called pollen xenia [1]. In many crops, pollen-donor selection can affect numerous important fruit traits including fruit set, size, shape, color, development time, and composition [2]. According to previous studies in apple [3] and grapes [4], xenia affects fruit quality and fruit seed weight. Xenia is also evident in kiwifruit, with the pollen donor affecting a series of important fruit traits including fruit weight, anthocyanin concentration, starch, fruit firmness, dry matter content, seed size and number, and titratable acid and ascorbic acid content. All these traits have been given attention in recent kiwifruit studies [5,6,7].

Kiwifruit belongs to the Actinidiaceae family, which includes 75 varieties and taxonomic units. There is extensive inter- and intraspecific ploidy variation in the *Actinidia*. All taxa appear to be dioecious and range in ploidy from diploid (2n = 2x = 58) to octoploid (2n = 8x = 232). It was reported that the pollen donor’s ploidy level affects the *A. arguta* fruits’ characteristics, including fruit weight, dry matter, flesh coloration, and nutritional components [8]. Many breeders will select suitable males for pollination with different ploidy pollen. Chen et al. detected that diploid (*A. chinensis*) males’ pollen positively influenced diploid (*A. chinensis*), tetraploid (*A. chinensis*), and hexaploid (*A. deliciosa*) female kiwifruit varieties [9]. However, pollen from hexaploid (*A. deliciosa*) males had a significantly more pronounced impact on homoploid female kiwifruit than on diploid (*A. chinensis*) and tetraploid (*A. chinensis*), respectively [10]. There was an increase in fresh weight but a decrease in well-developed seed number, lower seed quality, and delayed fruit maturity compared to the intraspecific homozygous pollination [10].

Although previous research on different species or different ploidy pollination schemes has been conducted, most focus on males that produce many seeds. In some kiwifruit cultivars, especially ‘Hayward’ (*A. deliciosa*, a variety widely cultivated worldwide), their eating quality, including taste and flavor, is highly affected by the seed numbers and size. Therefore, in this study, 10 different pollen donors, including four distant (*A. kolomikta*, *A. arguta*, *A. melanandra*, and *A. eriantha*) and two affinis (*A. chinensis* and *A. deliciosa*). were selected to investigate the xenia effect on the fruit quality of ‘Hayward’. Our research aims to reduce the production of seeds of ‘Hayward’ and further increase its fruit quality.

## 2. Results

### 2.1. Fruit-Setting Rate

The pollen viability of all tested donors ranged from 65.87% to 82.27% and met the trial requirements (Table 1). The fruit-set rates of the ten crossing combinations ranged from 20.55% to 99.33%. Six pollen donors from *A. chinensis* and *A. deliciosa* had a higher fruit-setting rate (>90.0%). In contrast, the more distantly related pollen donors, such as M7 (2x), M8 (4x), M9 (4x), and M10 (2x), had a significantly lower fruit-set rate, ranging from 20.55 to 37.32%, which did not meet the requirement of commercial production (fruit-set rate ≥ 80%). Therefore, these combinations were not included in the subsequent fruit quality analysis due to the lower fruit-setting rate.

### 2.2. Fruit and Seeds Appearance Trait

The average fruit weight derived from pollination with tetraploid and hexaploid pollen was significantly higher than that from diploid pollen. The same trend was observed for fruit transverse and longitudinal diameters (Table 2).

In terms of seed properties, M1 (2x) and M2 (2x) appeared to be good pollen sources for ‘Hayward’, greatly reducing the proportion of black seeds, the seeds’ transverse and longitudinal diameter, and the 1000-seed weight compared to other male plants used as pollinators (Table 2).

### 2.3. Fruit and Seed Characteristics and Seed Histology

**Figure 1:**
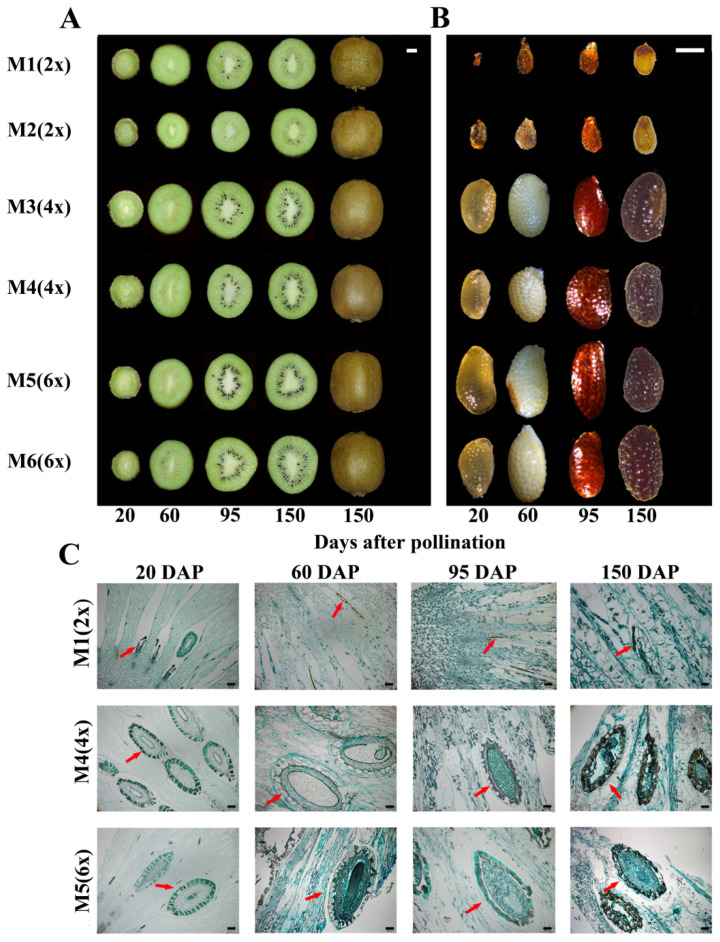
Fruit and seed characteristics and seed histology as affected by pollination by male plants of different ploidy. (**A**) ‘Hayward’ cut fruit sampled at four dates through the growing season; the scale bar is 1 cm; (**B**) seed characteristics at four dates through the growing season; the scale bar is 2 mm; (**C**) histological observations at four dates through the growing season, including the three pollinated males M1 (2x), M4 (4x), and M5 (6x); the red arrow points to the seeds, and the scale bars are 100 μm.

During fruit development, the seed number and size decreased greatly after pollination with a diploid pollen donor (Figure 1A,B). Microscopic characteristics of seeds from M1 (2x), M4 (4x), and M5 (6x) during development were further investigated (Figure 1C). Most of the seeds of M1 (2x)-pollinated fruits showed significant shrinkage at 20 DAP when compared to the M4 (4x) and M5 (6x) treatments. The seeds of fruits from M4 (4x) and M5 (6x) treatments had obvious inner and outer coat and bead center structures, the bead cell number was large, and the seeds were completely developed. At 60 DAP, the seeds of M1 (2x)-pollinated fruits had intact seed coats but few inclusions and severely degraded endosperm. This indicated that pollination with M1 (2x) resulted in marked seed abortion (Figure 1C). In contrast, regularly shaped embryos with obvious gaps between the embryo and the surrounding endosperm cells were observed in the seeds of M4 (4x)- and M5 (6x)-pollinated fruits. At 95 DAP, the seeds were already black in appearance and in the fruits obtained from M4 (4x) and M5 (6x) pollination, while the seed coat was still brown in the M1 (2x)-pollination treatment. Moreover, compared with M4 (4x) and M5 (6x), pollination with M1 (2x) resulted in the obvious hollowing of seeds. At 150 DAP, mature embryos were observed in both M4 (4x)- and M5 (6x)-pollinated fruit. Their seed coat was significantly thickened again compared to 95 DAP; in contrast, only the testa could be observed in the M1 (2x)-pollinated fruits.

### 2.4. Fruit Soluble Solid Content (SSC), Dry Matter Content (DM), Titratable Acidity (TA), Ratio of SSC and TA (SAR), and Ascorbic Acid (ASA) Content

Fruits pollinated with M4 (4x) and M6 (6x) had lower DM and SSC than those pollinated with other pollen donors (Table 3). In terms of TA, it was significantly higher in the fruits pollinated with M4 (4x) and M5 (6x) compared to fruits pollinated from the other pollen donors. The highest SAR and ASA were observed in M2 (2x)-pollinated fruit, reaching 18.72 and 68.91 ± 3.20 mg 100 g^−1^, respectively.

### 2.5. Sugar and Acid Components

As shown in Table 4, in the inner and outer pericarp, fructose, glucose, and total sugar content were the highest in M2 (2x)-pollinated fruit, resulting in the highest sweetness value (900.33 ± 14.72 and 811.85 ± 40.38, respectively). The acid content varied greatly between the inner and outer pericarp (Table 4). For the outer pericarp, M1 (2x) = pollinated fruits had the lowest malic acid (4.25 ± 0.15 mg g^−1^), citric acid (38.79 ± 0.53 mg g^−1^), and total acid (75.30 ± 1.78 mg g^−1^). The lower content of citric acid, quinic acid, and total acid was detected in the inner pericarp of M2 (2x)-pollinated fruits. Overall, for the outer and inner pericarp, the ratio of sweet/acid was higher in the fruit pollinated with the two diploid donors than in the fruits pollinated with the two tetraploid or two hexaploidy donors.

### 2.6. Volatile Compound Content in the Different Pollination Schemes

**Figure 2:**
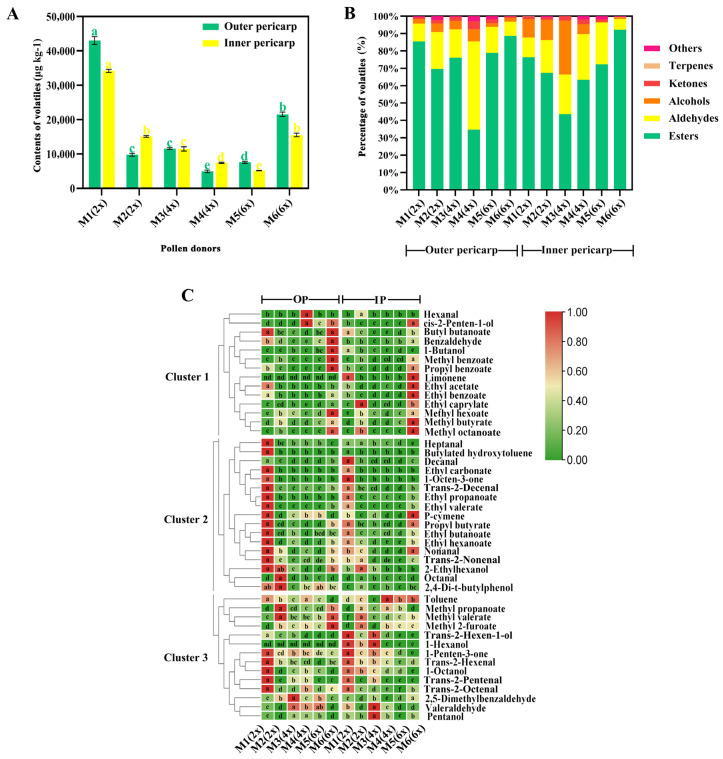
(**A**) Total volatile compounds in the outer and inner pericarp of fruits from different ploidy male plants treatments. (**B**) Percentage (%) of various compounds in the outer and inner pericarp of the fruits pollinated with different ploidy male plants. (**C**) Heatmap of volatile organic compounds content in the outer and inner pericarp of fruits pollinated with different ploidy male plants. Different lowercase letters indicate significant differences according to Duncan’s multiple range test at *p*-values < 0.05; nd indicates that the substance was not detected in these treatments. OP, outer pericarp; IP, inner pericarp.

The total amount of volatiles in the outer and inner pericarp after pollination with six different pollen donors is shown in Figure 2A. It was significantly higher in both the outer and inner pericarp of M1 (2x)-pollinated fruits and at a higher level in the inner pericarp after pollination with M2 (2x). Esters were the main volatile compound in the fruits from five pollen donors, followed by aldehydes or alcohols (Figure 2B). The only exception was the fruits from M4 (4x) pollination, which had a higher percentage of aldehydes.

A total of 45 volatile compounds, including 18 esters, 13 aldehydes, 7 alcohols, 2 ketones, and 5 others, were detected in the fruits pollinated with six different pollen donors (Figure 2C). All volatile compounds were divided into three main clusters according to their content levels in the outer and inner pericarp. In cluster 1, in general, the volatile compounds content of M6 (6x)-pollinated fruits was higher compared to the others except for hexanal and cis-2-Penten-1-ol, which were highest in the outer pericarp of M4 (4x)-pollinated fruits. Interestingly, in clusters 2 and 3, a higher volatile content was detected in the outer and inner pericarp of M1 (2x)- or M2 (2x)-pollinated fruits compared with fruits from other pollen donors except toluene, 2,5-dimethylbenzaldehyde, valeraldehyde, and pentanol.

### 2.7. PCA of Fruit Quality and E-Tongue and E-Nose Analysis

**Figure 3:**
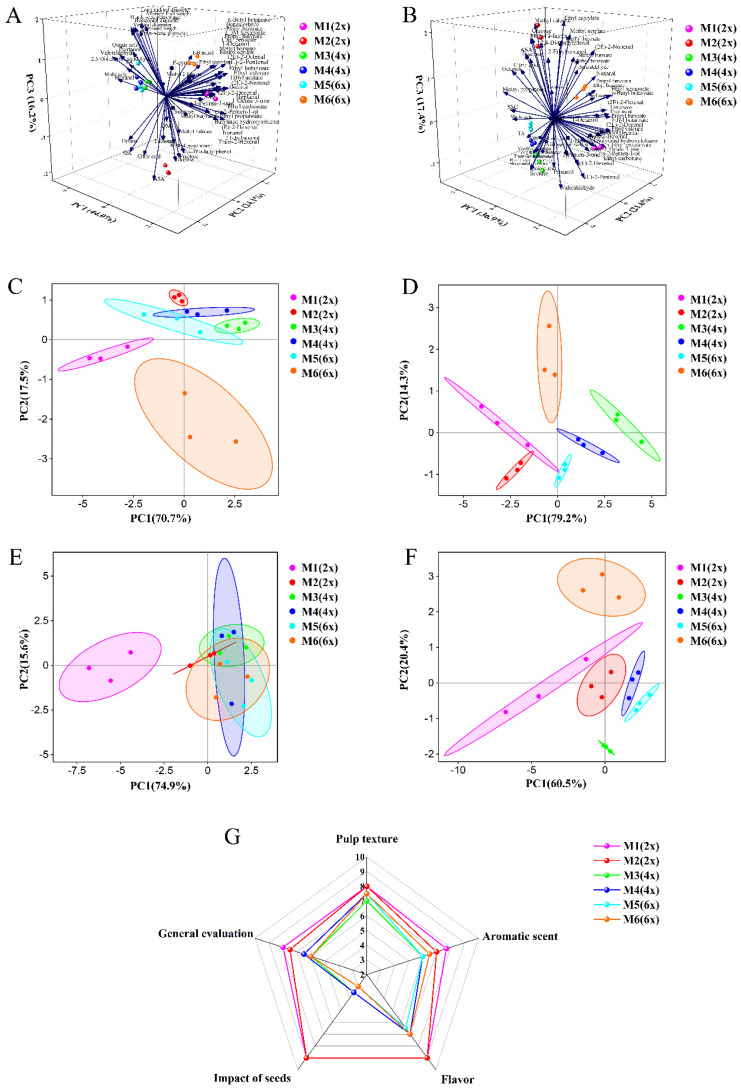
PCA of fruit quality and electronic tongue (E-tongue) and electronic nose (E-nose) evaluation of ‘Hayward’ outer pericarp (**A**,**C**,**E**), inner pericarp (**B**,**D**,**F**), and sensory evaluation (**G**) after pollination with different ploidy male plants. (**A**,**B**) Fruit quality principal component analysis (PCA) plot and loadings values; (**C**,**D**) PCA plot of E-tongue response sensory signals; (**E**,**F**) PCA plot of E-nose response sensory signals; (**G**) sensory evaluation radar chart.

To further explore how the six different pollen donors affected the overall fruit quality, a principal component analysis (PCA) was performed on the pooled fruit quality data measured in the outer and inner kiwifruit pericarp (Figure 3). In the outer pericarp, PC1, PC2, and PC3 explained 44.0%, 24.1%, and 16.2% of the variation, respectively (Figure 3A). The fruits from the six different pollen donors were classified into four groups: the M1 (2x)-, M2 (2x)-, and M6 (6x)-derived fruits were in a separate group, while M3 (4x), M4 (4x) and M5 (6x) were clustered in another group. Especially for the M1 (2x)- and M2 (2x)-derived fruits, the PCA clustering was obviously associated with the loading plots of glucose, fructose, citric acid, ASA, and most volatile compounds. Similar results were found for all tested samples’ inner pericarp quality data (Figure 3B). 

According to the E-tongue response signals, PCA analysis revealed that the first two principal components (PCs) explained 88.2% of the total variance in the outer pericarp of all samples. In particular, M1 (2x), M2 (2x), and M6 (6x) grouped separately from other pollen donors, which were overlapping (Figure 3C). In the inner pericarp of the fruit samples, the first and second PCs accounted for 93.5% of the total variance. Moreover, the M3 (4x), M4 (4x), M5 (6x), and M6 (6x) pollination treatments were clearly separated. There was an overlap among the two diploid donors, M1 (2x) and M2 (2x) (Figure 3D). Thus, these findings indicate that the overall taste of fruits derived from different pollination treatments, especially M1 and M2, was discriminated by E-tongue analysis.

In addition, the E-nose assay was used to determine volatile compounds in the fruit samples from the different pollen-donor treatments (Figure 3E,F). The first two PCs explained 90.5% and 80.9% of the total variance in the PCA analysis of quality traits in the outer and inner pericarp, respectively. In the outer pericarp, fruits from the M2, M3, M4, M5, and M6 pollen donors, overlapped together, and were not discriminated except for M1, which had a clear separation from other samples (Figure 3E). In contrast, in the inner pericarp, fruits from the six different pollen-donor treatments were grouped separately. Moreover, M1 and M2 were clustered together, similarly to M4 and M5, which formed another cluster (Figure 3F). Collectively, these observations suggest that significant differences could be detected in volatile compounds in fruits from different pollen-donor treatments using E-nose, especially in fruits from the two diploid donors.

Finally, to better investigate the fruit taste and flavor, sensory evaluation was employed (Figure 3G). As expected, fruits from the two diploid pollen donors (M1 (2x) and M2 (2x)) had higher scores in the overall evaluation. One reason is their better flesh texture, aromatic scent, and flavor. More importantly, regarding seeds affecting fruit taste and flavor, M1- and M2-pollinated fruits scored higher than others.

## 3. Discussion

The effect of pollination on fruit quality has been extensively studied in many fruit crops. In kiwifruit, most studies have focused on the effect of pollen parents on fruit quality to select the appropriate male plants for pollination [8,12,13]. The number of seeds and fruit size were important quality indexes. Furthermore, the number of seed set was usually positively correlated with the mature fruit size [14,15]. However, in recent years, there have been new consumer trends: seedless or little-seeded fruits are preferred by more and more consumers. However, this task is challenging, as the seed numbers and size affect fruit sensory quality.

‘Hayward’, a widely cultivated worldwide, is under great threat in China because of its big size and the high number of seeds in the fruit, which result in bad sensory traits. This study showed that six closely related pollen donors from *A. chinensis* and *A. deliciosa* contributed a higher fruit-setting rate (>90.0%), and only a few small seeds were found in the fruits from the two diploid donor M1 (2x) and M2 (2x) treatments, which is similar to the previous reports [9,10]. Fruit DM, SSC, TA, and SAR are important indexes for internal fruit quality evaluation [16]. Moreover, in this study, lower TA and higher SAR were observed in M1, M2, and M3. This indicates that fruit DM and SSC may share a weak relationship with the pollen donor’s ploidy.

Fruit sugar and acid contents determine the sweet and sour tastes, respectively, and have a major effect on kiwifruit sensory quality. The balance of the acid–sugar ratio primarily depends on the fruit’s citric acid content, which affects consumers’ acceptance of kiwifruit [17,18,19]. Similar to previous research [20,21], three sugars and three acid compounds were mainly detected in our study: sucrose, fructose, glucose, citric acid, quinic acid, and malic acid. The findings of this study suggest that the sweet and sour tastes of ‘Hayward’ were significantly affected by the ploidy of pollen donors. Diploid donors may contribute to a better balance between sweet and acidic taste in kiwifruit [22], while further research is needed on the mechanism.

The fruit aroma majorly contributes to consumers’ acceptance of the fruit due to its subtle mixture of volatile compounds [23]. Previously, esters and aldehydes have been reported as the predominant volatile compounds in edible kiwifruit [21,24,25,26], which corroborates with the findings of our study, and our study also found that the fruit aroma of ‘Hayward’ is affected by the ploidy of pollen donors as well as that of ‘Cuixiang’ and ‘Xuxiang’ [22].

## 4. Materials and Methods

### 4.1. Plant Materials

The experiment was carried out from 2021 to 2022 on mature vines of the ‘Hayward’ orchard in MeiXian, Shanxi Province, under standard management practices. The selection of ten different pollen donors is listed in Table 1. 

### 4.2. Pollen Preparation, Pollen Viability, and Pollination

Flowers at the popcorn stage, just prior to opening, were collected from the pollen-donor kiwifruit vines. The anthers were then separated and dried at room temperature for the pollen to be released. The collected pollen was stored at −30 °C in tubular glass vials with air-tight caps. Pollen viability was determined by an in vitro germination method according to Abreu with slight modifications [27]. The modified medium contained 10% sucrose, 100 mg/L boric acid, and 10 mg/L calcium nitrate and was solidified with 0.8% agar. After incubation for 3.5 h in a constant temperature incubator set at 28 °C and 100 rpm, the pollen grains were placed under a light microscope for observation. 

Ten pollination treatments were designed and conducted (Table 1). For flowers, 10 fruit branches from each tree were selected from 5 trees of ‘Hayward’. All shoots were bagged before flowering to avoid accidental cross-pollination. Newly opened flowers were hand-pollinated by inverting the vial containing the dried anthers and pollen over the pistils. Bags were resealed immediately after pollination and removed after the fruit set. After 20 days of pollination, the fruit-setting rate was recorded and calculated according to method of Gharaghani [28].

### 4.3. Observation of Fruit and Seed Characteristics and Histological Analysis of Seed Development

The developing fruits of six combinations with higher fruit-set rates (>90.0%) were collected randomly on each of the 10 treated vines at 20, 60, 95, and 150 days after pollination (DAP). The collected samples were photographed with a camera (Nikon D750, Shanghai, China) to record fruit growth and development, and a stereomicroscope (MZ10F, Vizsla, Germany) was used to observe seed changes.

The fruits of M1 (2x), M4 (4x), and M5 (6x) were further selected during development to investigate the seeds’ microstructure. The samples were embedded with paraffin according to the method of [29] and were then cut into 10–12 µm thick sections using a microtome (RM2235, Shanghai, China). Ribbons were placed on glass slides, and the slides were dried at 37 °C on a warming plate. The slides were deparaffinized in xylene for 30 min (twice), then hydrated in a graded ethanol series (100, 95, 85, 70, and 50% in DW). All preparations sections were stained with 1% fast green for 1 min at room temperature. Finally, the sections were observed and imaged using a microscope (BX51+IX71, Olympus, Tokyo, Japan) under conventional bright-field illumination. 

### 4.4. External Evaluation of Fruit and Seed Quality

At 150 DAP, all the sampled fruits with a minimum SSC of 6.5% were harvested. The fresh weight (FW) was recorded using the electronic balance, and the fruit dimensions were measured with vernier calipers. To assess the seed number and weight, the seeds were collected from 12 kiwifruits for each pollination treatment when they were ripening (firmness 8–10 N) [22]. The collected kiwifruit seeds were properly washed, dried, and counted, and the percentage of black seeds was recorded. Then, 1000 randomly selected seeds from each fruit were counted and weighed. Thirty seeds were randomly selected from each treatment, and their transverse and longitudinal diameters were calculated using ImageJ software.

### 4.5. Determination of Fruit Soluble Solid Content (SSC), Dry Matter Content (DM), Titratable Acidity (TA), and Ascorbic Acid (ASA) Content

When the fruits reached an edible state (firmness at 8–10 N), kiwifruit samples were meshed into juice, and the SSC was determined with a handheld refractometer (PAL-1, Atago Co., Ltd., Minato-ku, Tokyo, Japan). The DM% of an equatorial fruit section with a 2–3 mm thickness was measured after drying at 60 °C for 24 h [30]. TA was determined according to the method of [31]. The kiwifruit mesocarp (1 g) was diluted with 3 mL of distilled water, and the mixture was filtered (0.22 µm pore size membrane filter). Then, 3 mL of the filtrate was titrated with 0.1 mol L^−1^ NaOH. TA was calculated with the formula of [32] and was expressed as the citric acid concentration (%). The ASA content was determined by 2,6-dichlorophenol according to [33].

### 4.6. Determination of Soluble Sugars and Organic Acids in Fruits

Soluble sugars and organic acids determination was carried out according to the method of [34] with minor modifications. Specifically, about 0.1 g of fruit sample was placed in a 2 mL lyophilization tube, and 1400 µL 75% methanol (pre-chilled at −20 °C) and 100 μL Ribitol (400 ppm) were added. The samples were vortexed for 30 s, shaken at 70 °C for 30 min at 950 rpm, and centrifuged at 11,000 rpm for 10 min. The supernatant was transferred to centrifuge tubes, and 750 µL trichloromethane (CHCl_3_) was added, followed by 1400 µL ddH_2_O (stored at 4 °C), and vortexed for 10 s and centrifuged at 2200 rpm for 15 min. Next, 5 µL of the supernatant was transferred to a 2 mL centrifuge tube and dried for 4 h in a vacuum concentrator. Then, 40 µL of derivatization reagent (pyridine solution of methoxamine hydrochloride, 20 mg mL^−1^) was added after evaporation and derivatization at 37 °C for 2 h. Then, 60 µL of N-methyltrimethylsilyl trifluoroacetamide (MSTFA) was added, followed by derivatization at 37 °C for 30 min. After slight centrifugation, the samples were transferred to the upper sample bottle and detected using GC-MS (QP2010, Shimadzu, Osaka, Japan) [31].

### 4.7. Determination of Volatile Substance Content

The determination of volatile organic compounds was carried out according to [19]. Briefly, 1 g of the fruit powder was placed in a 30 mL glass bottle, with 2 mL of saturated NaCl solution and 5 µL of internal standard (cyclohexanone) added. Then, a 65 µm PDMS/DVB SPME fiber (Supelco, Darmstadt, Germany) was exposed to the vial headspace for 30 min at 45 °C with continuous agitation. Headspace solid-phase microextraction (HS-SPME) and gas chromatography–mass spectrometry (7890B-5875C, GC-MS, Agilent, Palo Alto, CA, USA) were used to determine the volatile compounds of the samples [35]. The extractor needle was then inserted into the sample bottle and was extracted for 30 min. Then, the extractor was inserted into the inlet of GC for analysis. GC-MS conditions were as follows: high-purity helium as the carrier gas, flow rate 1.0 mL min^−1^; programmed temperature rise: 40 °C for 3 min, then 3 °C min^−1^ to 100 °C, then 5 °C min^−1^ to 245 °C; no split injection. Mass spectrometry conditions: ion source temperature 230 °C; electron energy 70 eV; scan range 35–350 *m/z*. Qualitative analysis was performed using the NIST 2014 MS library search with RIs on HP-5 ms columns. The relative contents of the detected volatiles were calculated using peak area normalization.

### 4.8. E-Tongue and E-Nose Analysis

E-tongue and E-nose analysis was performed according to [31]. For e-tongue measurements, frozen kiwifruit samples were ground with a grinder in liquid nitrogen to obtain uniformly sized particles. Then, 100 mL of purified water was added to 0.02 g of samples and centrifuged, and the resulting supernatant (flavor compound) was poured into a test cup for electron tongue measurement. An Astree E-tongue (Alpha M.O.S, Toulouse, France) comprised of seven sensors was used to collect data. Each sample was analyzed seven times, with each analysis period being 120 s. The value generated at 120 s was used as the final output value. All measurements were taken at room temperature (25 °C).

An E-nose (WMA Airsense Analytics GmbH, Schwerin, AV, Germany) with ten different gas sensors were used in this study. Kiwifruit juice (3 mL) was placed in a 40 mL glass vial for headspace accumulation. The glass vial was sealed and then maintained at 25 °C for 20 min. Subsequently, one needle with a carbon filter was inserted 5 cm below the glass vial surface for balancing gas. The other needle with a 0.22 µm membrane filter was inserted 3 cm below the glass vial surface to determine the odor signal. Other analytical parameters were as follows: clearing time, 60 s; measurement process, 150 s; waiting time, 5 s; cavity air fluid, 0.4 L min^−1^; injection air fluid, 0.016 L min^−1^. All E-nose measurements were carried out in a room with a controlled temperature (25 °C) and relative air humidity (70%).

### 4.9. Sensory Evaluation

Sensory evaluation was according to a previous method [36] with minor modifications. Twelve sensory evaluators were selected to conduct the sensory evaluation of ‘Hayward’ fruits from six different ploidy-treated male plants when they reached the edible state (8–10 N) in five aspects: flesh texture, aroma, taste, the impact of seeds, and overall evaluation (Table S1). Higher scores indicated that the sample had stronger quality sensory characteristics, and lower scores corresponded to weaker sensory characteristics. The results were standardized and analyzed. The panel candidates were recruited from the College of Horticulture at Northwest A&F University. Using a questionnaire, they were preliminarily selected based on their personal information, including health status, sensory acuity, and familiarity with the fruit.

### 4.10. Statistical Analyses

The experimental data were statistically analyzed and processed with Microsoft Excel 2010 and SPSS 22.0 (Armonk, NY, USA: IBM Corp.). Duncan’s method detected the differences in all pollination combinations at *p* ≤ 0.05. Heatmap and principal component analysis (PCA) was performed using origin 2019 (Informer Technologies, Inc., https://www.informer.com/). 

## 5. Conclusions

In this study, the different pollen donors with different species and ploidy levels significantly affected the fruit-setting rate, seed number and weight, and fruit quality of ‘Hayward’. It is difficult for an individual pollen donor to achieve excellent performance on all indicators. However, it was noteworthy that the diploid (from the related species *A. chinensis*) pollination donors gave the ‘Hayward’ seedless fruit a better taste and flavor than tetraploid (*A. chinensis*) or hexaploid (*A. deliciosa*) donors. In terms of fruit size and weight, effective cultivation measures should be performed to obtain fruits with normal size and weight. Overall, our findings provide new insights towards further improvement of ‘Hayward’ and producing other seedless kiwifruit fruits.

## Figures and Tables

**Table 1:** The species, ploidy level, and pollen vitality of different pollen donors and fruit-setting rate after pollination.

Pollen Donors	Species	Ploidy Level	Name	Pollen Vitality (%)	Flowers-Number	Fruit Number	Fruit-Setting Rate (%)
M1 ^a^	*A. chinensis*	Diploid, 2x	M1 (2x)	77.46	200	196	98.00 a
M2 ^a^	*A. chinensis*	Diploid, 2x	M2 (2x)	76.21	218	201	92.20 c
M3 ^a^	*A. chinensis*	Tetraploid, 4x	M3 (4x)	82.27	150	149	99.33 a
M4 ^b^	*A. chinensis*	Tetraploid, 4x	M4 (4x)	70.35	171	162	94.74 b
M5 ^a^	*A. deliciosa*	Hexaploid, 6x	M5 (6x)	76.13	163	153	93.87 bc
M6 ^a^	*A. deliciosa*	Hexaploid, 6x	M6 (6x)	82.13	193	180	93.26 bc
M7 ^a^	*A. kolomikta*	Diploid, 2x	M7 (2x)	66.33	154	38	24.68 f
M8 ^a^	*A. arguta*	Tetraploid, 4x	M8 (4x)	75.79	146	30	20.55 g
M9 ^b^	*A. melanandra*	Tetraploid, 4x	M9 (4x)	68.05	142	53	37.32 d
M10 ^b^	*A. eriantha*	Tetraploid, 4x	M10 (4x)	65.87	40	11	27.50 e

Note: ^a^ represents the material from MeiXian, Shanxi Province; ^b^ represents the material from Wuhan city, Hubei Province. Different lowercase letters after data in the same column indicate significant differences (*p* < 0.05). The following table is the same.

**Table 2:** Effects of the six selected pollen donors on fruit and seed traits of ‘Hayward’.

Male	Fruit			Seeds			
	Average Fruit Weight (g)	Longitudinal Diameter (mm)	Transverse Diameter (mm)	Percentage of Black Seed	Horizontal Diameter (mm)	Vertical Diameter (mm)	1000-Seed Weight (g)
M1 (2x)	69.81 ± 6.67 c	49.73 ± 2.50 c	51.19 ± 3.16 c	13.95%	0.646 ± 0.146 c	0.972 ± 0.061 c	0.21 e
M2 (2x)	68.12 ± 6.17 c	48.44 ± 2.01 c	52.39 ± 2.81 c	12.50%	0.622 ± 0.142 c	1.030 ± 0.182 c	0.24 d
M3 (4x)	104.94 ± 8.92 b	57.17 ± 3.25 b	67.11 ± 3.51 b	100%	1.429 ± 0.138 ab	2.457 ± 0.133 a	1.07 c
M4 (4x)	104.68 ± 10.97 b	56.69 ± 2.07 b	68.75 ± 3.54 ab	100%	1.317 ± 0.107 b	2.265 ± 0.178 b	1.06 c
M5 (6x)	113.53 ± 10.58 a	57.67 ± 2.51 ab	68.68 ± 3.31 ab	100%	1.394 ± 0.102 ab	2.438 ± 0.148 a	1.68 a
M6 (6x)	120.91 ± 7.83 a	59.24 ± 3.27 a	69.65 ± 2.44 a	100%	1.464 ± 0.092 a	2.532 ± 0.218 a	1.54 b

Note: different lowercase letters after data in the same column indicate significant differences (*p* < 0.05).

**Table 3:** The intrinsic fruit quality from different pollen donors.

Male	DM (%)	SSC (%)	TA (%)	SAR	ASA (mg/100 g)
M1 (2x)	20.22 ± 0.97 ab	15.1 ± 0.88 b	0.97 ± 0.07 b	15.60 ± 0.47 b	52.92 ± 2.41 bc
M2 (2x)	20.98 ± 1.05 a	17.6 ± 0.64 a	0.94 ± 0.23 b	18.72 ± 0.68 a	68.91 ± 3.20 a
M3 (4x)	20.35 ± 1.09 ab	17.3 ± 0.12 a	0.93 ± 0.00 b	18.60 ± 0.08 a	52.57 ± 2.00 bc
M4 (4x)	19.16 ± 0.89 c	15.5 ± 0.74 b	1.47 ± 0.17 a	11.09 ± 0.30 c	51.18 ± 2.64 c
M5 (6x)	20.54 ± 0.72 a	17.0 ± 0.39 a	1.74 ± 0.18 a	10.03 ± 1.15 c	56.65 ± 1.94 b
M6 (6x)	19.51 ± 0.73 bc	13.6 ± 0.11 c	1.06 ± 0.29 b	12.01 ± 1.02 c	45.26 ± 0.26 d

Note: different lowercase letters after data in the same column indicate significant differences (*p* < 0.05).

**Table 4:** Contents of sugars and organic acid compounds in the outer pericarp and inner pericarp of fruits pollinated with different pollen donors.

Male	Fructose (mg g^−1^)		Glucose (mg g^−1^)		Sucrose (mg g^−1^)		Total Sugar (mg g^−1^)		Sweetness Value	
	Outer Pericarp	Inner Pericarp	Outer Pericarp	Inner Pericarp	Outer Pericarp	Inner Pericarp	Outer Pericarp	Inner Pericarp	Outer Pericarp	Inner Pericarp
M1 (2x)	21.08 ± 0.06 b	19.15 ± 0.45 bc	27.98 ± 0.73 cd	26.68 ± 0.75 c	27.30 ± 2.71 a	20.08 ± 1.22 b	76.36 ± 3.18 b	65.91 ± 2.11 b	83.78 ± 2.99 b	72.27 ± 2.24 b
M2 (2x)	25.08 ± 0.50 a	21.86 ± 0.97 a	38.91 ± 0.60 a	36.06 ± 1.47 a	18.90 ± 0.97 b	17.67 ± 1.71 b	82.89 ± 1.39 a	75.61 ± 3.78 a	90.03 ± 1.47 a	81.19 ± 4.04 a
M3 (4x)	20.98 ± 0.44 b	19.38 ± 0.44 bc	29.42 ± 0.63 bc	27.09 ± 0.61 c	20.84 ± 1.10 b	19.98 ± 0.39 b	72.43 ± 1.45 b	70.11 ± 1.15 ab	78.15 ± 2.22 b	72.85 ± 1.54 b
M4 (4x)	20.03 ± 0.62 b	18.77 ± 0.04 c	26.65 ± 0.82 d	25.35 ± 0.06 c	25.75 ± 0.52 a	25.98 ± 1.15 a	71.24 ± 2.09 b	66.44 ± 1.39 b	79.46 ± 1.63 b	76.57 ± 1.11 ab
M5 (6x)	21.30 ± 0.14 b	20.77 ± 0.61 ab	29.89 ± 0.19 b	29.99 ± 0.75 b	13.36 ± 0.94 c	18.11 ± 0.44 b	64.55 ± 0.66 c	68.87 ± 1.80 ab	71.56 ± 0.63 c	75.45 ± 2.04 ab
M6 (6x)	20.52 ± 0.11 b	18.83 ± 0.01 c	27.24 ± 0.09 d	26.10 ± 0.06 c	13.14 ± 0.44 c	13.30 ± 0.91 c	60.91 ± 0.41 c	58.22 ± 0.98 c	68.12 ± 0.44 c	64.51 ± 0.97 c
	Malic acid (mg g^−1^)		Citric acid (mg g^−1^)		Quinic acid (mg g^−1^)		Total acid (mg g^−1^)		Sweet/acid ratio	
	Outer pericarp	Inner pericarp	Outer pericarp	Inner pericarp	Outer pericarp	Inner pericarp	Outer pericarp	Inner pericarp	Outer pericarp	Inner pericarp
M1 (2x)	4.25 ± 0.15 c	5.22 ± 0.41 c	38.79 ± 0.53 b	50.21 ± 0.16 bc	32.26 ± 1.14 bc	19.29 ± 0.21 bc	75.30 ± 1.78 b	74.71 ± 0.78 bc	1.11 ± 0.04 a	0.97 ± 0.03 b
M2 (2x)	6.84 ± 0.16 b	5.87 ± 0.21 c	47.79 ± 0.50 a	44.48 ± 0.29 c	28.91 ± 0.29 c	18.21 ± 3.30 c	83.55 ± 0.36 ab	68.56 ± 3.55 c	1.08 ± 0.01 a	1.18 ± 0.02 a
M3 (4x)	6.78 ± 0.62 b	7.28 ± 0.04 b	42.70 ± 3.03 ab	61.24 ± 1.95 a	33.51 ± 2.51 bc	23.58 ± 0.77 ab	83.00 ± 4.25 ab	92.10 ± 2.71 a	0.87 ± 0.03 bc	0.79 ± 0.01 c
M4 (4x)	9.15 ± 1.21 a	10.54 ± 0.76 a	41.94 ± 2.40 ab	57.96 ± 0.51 ab	40.80 ± 1.55 a	25.28 ± 0.51 a	91.89 ± 7.07 a	93.79 ± 0.90 a	0.95 ± 0.05 b	0.82 ± 0.01 c
M5 (6x)	9.21 ± 0.33 a	8.17 ± 0.52 b	41.98 ± 0.36 ab	50.74 ± 1.76 bc	37.22 ± 1.99 ab	21.14 ± 0.46 abc	88.41 ± 3.11 ab	80.06 ± 1.81 b	0.81 ± 0.02 c	0.94 ± 0.02 b
M6 (6x)	6.80 ± 0.02 b	5.66 ± 0.37 c	39.51 ± 2.37 b	54.50 ± 5.36 ab	32.43 ± 0.89 bc	20.14 ± 0.16 bc	78.74 ± 3.63 ab	80.30 ± 5.40 b	0.89 ± 0.03 bc	0.81 ± 0.06 c

Note: sweetness value = sucrose content × 1.00 + fructose content × 1.75 + glucose content × 0.70, sweet-acid ratio = sweetness value/total acid content [11]. Different lowercase letters after data in the same column indicate significant differences (*p* < 0.05).

## Data Availability

The data used in this manuscript will be made available from the corresponding authors upon reasonable request.

## Supplementary Materials

The supporting information can be downloaded at: https://www.mdpi.com/article/10.3390/ijms24108876/s1.
