# Supplementary material for: The Pollen Donor Affects Seed Development, Taste, and Flavor Quality in ‘Hayward’ Kiwifruit
Source: Int J Mol Sci. 2023 May 17;24(10):8876. doi: 10.3390/ijms24108876 (PMC10219053; doi:10.3390/ijms24108876)
Supplement: Supplementary file 1 [file ijms-24-08876-s001.zip › ijms-2314654-SI.pdf]

Supplementary Materials

Table S1. Sensory evaluation criteria.

sensory characteristics	Scoring criteria				
	0-2	3-4	5-6	7-8	9-10
Pulp Texture	Hard pulp	Less juice and harder pulp	More refreshing	Juicy and crisp	Juicy, fine texture and crispness
Aromatic scent	Bad aroma	No aroma	Lighter aroma	Stronger aroma	Strong aroma
Flavor	Obvious odor	Faint odor	lighter	More sweet and sour	Sweet and sour
Whether seeds affect the taste	Severe effects	Effects	Moderate	slightly	No effect
